# Photographic Panorama of the World’s Fair

**Published:** 1894-04

**Authors:** 


PHOTOGRAPHIC PANORAMA OF THE WORLD'S FAIR.

Published by Mast, Crowell & Kirkpatrick, Springfield, Ohio. Showing pictures of the grand buildings, of glittering domes, of massive arches, of noble statuary, of jetting fountains, of beautiful interior exhibits, of Venetian gondolas, gliding over the deep lagoons, of pavilions, of foreign villages, of cafés, of the wooded island and many other attractions of the Dream City and the Midway Plaisance. We have received three parts of this interesting publication. Those who went to the Fair will feel again the pleasure they did there, and those who did not will find herein a source of interest and education. Price and information may be obtained by applying to the publishers. Each part contains 55 photographic views.

